# Tony (Anthony James) Pawson, a giant in the field of cell signaling research, dies unexpectedly at the age of 60

**DOI:** 10.1186/1478-811X-11-61

**Published:** 2013-08-19

**Authors:** Stephan M Feller

**Affiliations:** 1 Section Tumor Biology, Institute for Molecular Medicine, ZAMED, Martin-Luther-University Halle-Wittenberg, Halle (Saale), Germany; 2 Biological Systems Architecture Group, Weatherall institute of Molecular Medicine, University of Oxford, Oxford, United Kingdom

## Abstract

Tony was widely recognized as a world leader in the field of signal transduction and conducted seminal work especially on signaling processes related to protein – protein interactions and cancers. He will be much missed by many colleagues around the world.

## Maintext

Tony obtained his MA in Biochemistry from Cambridge, working with Tim (Richard Timothy) Hunt (Nobel Laureate 2001), and then a PhD from King’s College London in 1976 for research conducted in Alan Smith’s group. From 1976–1980 Tony was a postdoc at UC Berkeley, working with Peter Duesberg and G. Steven Martin; and from 1981–1985 he was an assistant professor at University of British Columbia, Vancouver, Canada.

In 1985, Tony moved to the Samuel Lunenfeld Research Institute at Mount Sinai Hospital, Toronto, Canada, where he established a firm base, becoming a distinguished scientist and director of research, as well as a Professor of Molecular Genetics at the University of Toronto.

Tony received numerous science awards, including, in 2010, the first STS/CCS Honorary Medal, jointly sponsored by the Signal Transduction Society (http://www.sigtrans.de) and CELL COMMUNICATION AND SIGNALING [1] (see http://www.biosignaling.com for further information; see also Picture). Furthermore, Tony was a member of numerous editorial boards, including that of CELL COMMUNICATION AND SIGNALING from 2008 onwards.

**Figure:**
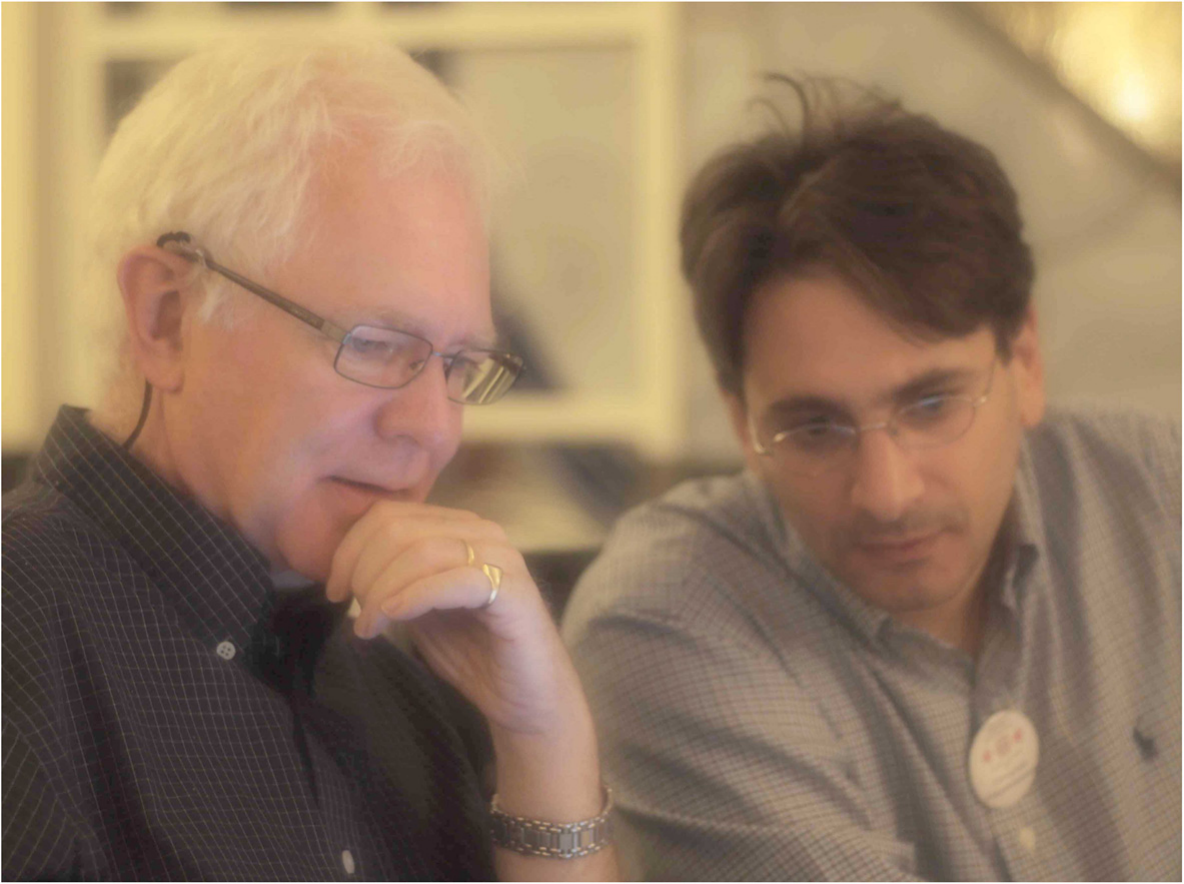
**Tony discussing results with Panagis Filippakopoulos at the 2010 STS conference in Weimar, Germany.** Tony was the inaugural recipient of the STS/CCS Honorary Medal in that year. Photo kindly provided by Toby Gibson, EMBL.

Throughout much of his career, Tony was feared as a highly effective competitor who not only came up with his own innovative leads but also recognized other promising new routes in signaling research with great efficacy and swiftly engaged in creative research on these.

I learned this first hand during my own PhD thesis research. Tony attended a talk in New York City, where my PhD supervisor, Hidesaburo Hanafusa [2], presented, for the first time, my research results in public [3]. Within two years, Tony and his collaborators had a manuscript in *Nature* in press on this topic [4].

At the same time, Tony was also a generous mentor and enthusiastic collaborator who shaped and led the world’s research on protein interaction domains and protein phosphorylation in many ways. A vast number of scientists have directly or indirectly benefited from his guidance as well as his scientific breakthroughs.

Tony’s greatest scientific achievement, among many, is arguably the discovery of Src Homology 2 (SH2) domains in the mid 80s, which became the archetypal domain family for the study of protein – protein interactions. At the 1990 Oncogene Meeting in Frederick, Maryland, Tony, together with Bruce Mayer (then in David Baltimore’s group) and the Hanafusa team, was also the amongst first to report that SH2 domains interact with tyrosine-phosphorylated protein epitopes, thereby identifying protein modification as a key tool in the arsenal of signaling cells. This research area has flourished ever since and today literally hundreds of protein modifications are known, which impact on virtually all aspects of cell regulation phenomena.

Tony’s unassuming personality, sharp intellect, valuable advice and openness for principal investigators and students alike will remain a treasured memory for all who had the privilege to closely interact with him.
